# Electronic Health Record Use During Paid Time Off Among Primary Care Physicians

**DOI:** 10.1001/jamanetworkopen.2025.0465

**Published:** 2025-03-11

**Authors:** Corey Obermiller, Richa Bundy, Lauren Witek, Adam Moses, Lindsey Carlasare, Gary Rosenthal, Christine Sinsky, Ajay Dharod

**Affiliations:** 1 Informatics and Analytics, Department of Internal Medicine, Wake Forest University School of Medicine, Winston-Salem, North Carolina; 2 General Internal Medicine, Department of Internal Medicine, Wake Forest University School of Medicine, Winston-Salem, North Carolina; 3 Department of Implementation Science, Division of Public Health Sciences, Wake Forest University School of Medicine, Winston-Salem, North Carolina; 4 Wake Forest Center for Healthcare Innovation, Wake Forest University School of Medicine, Winston-Salem, North Carolina; 5 Wake Forest Center for Artificial Intelligence, Wake Forest University School of Medicine, Winston-Salem, North Carolina; 6 American Medical Association, Chicago, Illinois

## Abstract

This cohort study uses electronic health record data to quantitatively assess primary care physicians’ work during paid time off.

## Introduction

Burnout is increasingly prevalent among primary care physicians (PCPs) and is linked to electronic health record (EHR) use.^1,2,3,4^ Thus, the importance of organizational efforts to support fully disengaging from work-related activities during vacation could enhance patient care quality and reduce costs.^5^ However, a national survey^6^ of 3024 physicians across specialties between 2020 and 2021 found that 70.4% worked during vacation days. Additionally, 59.6% reported taking 15 or fewer vacation days in the previous year. Physicians cited finding clinical coverage, financial impact, and EHR inbox volume as barriers to taking vacations.^6^ Building on these findings, our study uses objective EHR data to quantitatively assess PCPs’ work during paid time off (PTO).

## Methods

We conducted a retrospective cohort study of PCPs within a large academic and community health care system, analyzing EHR user action logs from January 2022 to July 2023. The study was approved by the institutional review board of Wake Forest University School of Medicine, which granted a waiver of informed consent due to the retrospective and deidentified nature of the data collected, in accordance with 45 CFR §46. Results were reported in accordance with STROBE reporting guidelines.

Physicians were eligible if they worked in family or internal medicine clinics, with community physicians at 1.0 clinical full-time equivalent (FTE) and academic physicians at greater than or equal to 0.5 FTE. PTO data were obtained from administrative records for academic physicians and derived from EHR scheduling data for community physicians. For each vacation, we created PTO blocks defined as 2 consecutive PTO weekdays, including their adjacent or inclusive weekends. PTO blocks were classified as short (2-5 days), medium (6-10 days), or long (>10 days), with each day within a block further categorized as the first, middle, or last day. The median time spent in the EHR per day was calculated for each physician and summarized using descriptive statistics. Kruskal-Wallis tests for association of EHR use metrics across length and day types were performed. Statistical significance was defined as 2-sided *P* < .05.

## Results

Among the 56 PCPs (33 male [58.9%]; mean [SD] age, 50.4 [12.2] years) (Table) the median (IQR) time in the EHR per PTO day was 16.1 (6.8-26.2) minutes, with a median (IQR) of 39.0% (25.8%-57.5%) of days having at least some EHR use and 19.0% (5.8%-26.5%) experiencing over 30 minutes per day. Longer lengths of PTO blocks were associated with decreased EHR engagement (χ^2^_2_ = 12.84; *P* = .002). Specifically, physicians spent a median (IQR) of 50.0% (31.0%-75.0%) of short vacation days and 18.0% (10.5%-45.0%) of long vacation days with some EHR use. EHR engagement also increased at the beginning and end of vacations (χ^2^_2_ = 17.12; *P* < .001). Specifically, physicians spent a median (IQR) of 57.0% (25.0%-77.8%) of first days and 63.5% (36.0%-97.2%) of last days in the EHR. In comparison, physicians spent only a median (IQR) of 29.0% (15.5%-54.2%) of middle days in the EHR (Figure). When investigating EHR tasks performed during PTO, we found that physicians spent a median (IQR) of 39.5% (29.8%-54.2%) of total EHR time performing inbox-related tasks.

**Table.** Characteristics of Physicians in the Cohort

Characteristic	Physicians, No. (%) (N = 56)
Sex	
Female	23 (41.1)
Male	33 (58.9)
Specialty	
Family medicine	35 (62.5)
Internal medicine	21 (37.5)
Type	
Community physicians	32 (57.1)
Physicians with academic responsibilities	24 (42.9)
Age, y	
Mean (SD)	50.4 (12.2)
Median (IQR)	49.0 (40.0-63.0)
PTO blocks per year (vacations), No.	
Mean (SD)	5.7 (3.1)
Median (IQR)	5.3 (3.8-6.8)
Time in EHR on PTO days, min	
Mean (SD)	18.9 (16.9)
Median (IQR)	16.1 (6.8-26.2)
Percentage of days in the EHR during PTO	
Mean (SD)	42.8 (25.0)
Median (IQR)	39.0 (25.8-57.5)
Time in EHR on standard workdays, min	
Mean (SD)	221.7 (63.8)
Median (IQR)	229.1 (167.9-266.4)

Abbreviations: EHR, electronic health record; PTO, paid time off.

**Figure.**
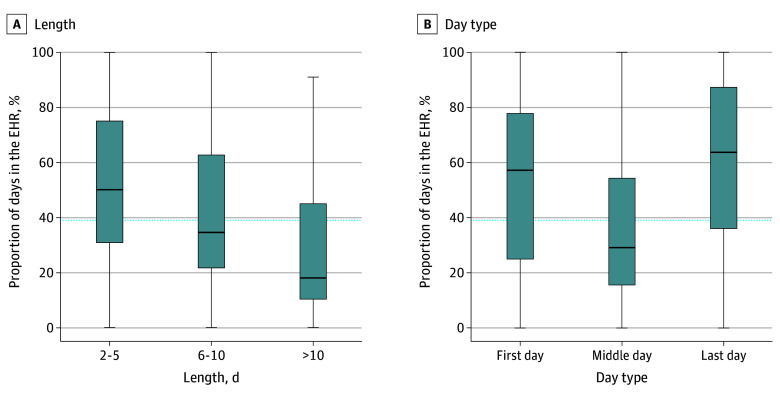
Percentage of Days in the Electronic Health Record (EHR) Across Length and Day Type Longer vacations were associated with lower EHR engagement rates (A). The first and last days of a vacation had higher rates of EHR use in comparison to the middle days of a vacation (B). The blue dotted line represents the overall median percentage of days in the EHR at 39%. Lines within boxes denote medians, tops of boxes denote 75th percentiles, bottoms of boxes denote 25th percentiles, and whiskers denote minimum and maximum values.

## Discussion

This cohort study found that PCPs often engage in EHR activities during PTO, particularly at the start and end of longer vacations, reflecting challenges in fully disconnecting from work. Longer PTO blocks were associated with reduced EHR use, suggesting that extended vacations enable better disengagement. Notably, no significant differences in EHR use were observed between academic and community physicians despite differing workloads.

This study has limitations. PTO data were directly available only for academic physicians, whereas vacation periods for community physicians were estimated using a proxy measure. Including weekends at both ends of PTO blocks may have overestimated their length, although sensitivity analyses showed no significant association.

Organizations should implement strategies to minimize clinical tasks during PTO. Future research should explore interventions that help physicians fully disconnect from work.

## Notes

Supplement.
Data Sharing Statement
