# Supplementary material for: Electronic Health Record Use During Paid Time Off Among Primary Care Physicians
Source: JAMA Netw Open. 2025 Mar 11;8(3):e250465. doi: 10.1001/jamanetworkopen.2025.0465 (PMC11897832; doi:10.1001/jamanetworkopen.2025.0465)
Supplement: Supplement. — Data Sharing Statement [file jamanetwopen-e250465-s001.pdf]

Data Sharing Statement

Obermiller. Electronic Health Record Use During Paid Time Off Among Primary Care Physicians. *JAMA Netw Open*. Published March 11, 2025. doi:10.1001/jamanetworkopen.2025.0465

Data
Data available: No
